# NIPAL1 Drives a Metabolic‐Epigenetic Feedback Loop to Promote Lactate‐Mediated Immune Evasion in Esophageal Cancer

**DOI:** 10.1002/advs.202520055

**Published:** 2026-03-12

**Authors:** Ri‐Xin Chen, Xiao‐Dan Ma, Shui‐Dan Xu, Ao‐Tian Mo, Min‐Hua Deng, Jun‐Han Wu, Wei‐Tao Zhuang, Jin‐Ling Duan, Shu‐Huan Xie, Jin‐Long Lin, Peng Lin, Ji‐Ming Tang, Hai‐Yu Zhou, Xiao‐Song Ben, Gui‐Bin Qiao, Dan Xie

**Affiliations:** ^1^ Department of Thoracic Surgery Guangdong Provincial People's Hospital (Guangdong Academy of Medical Sciences) Southern Medical University Guangzhou China; ^2^ State Key Laboratory of Oncology in South China Guangdong Provincial Clinical Research Center for Cancer Sun Yat‐sen University Cancer Center Guangzhou China; ^3^ Medical Research Institute Guangdong Provincial People's Hospital (Guangdong Academy of Medical Sciences) Southern Medical University Guangzhou China; ^4^ Department of Radiology Sun Yat‐sen University Cancer Center Guangzhou Guangdong China; ^5^ Department of Urology The First Affiliated Hospital of Guangzhou Medical University Guangzhou China; ^6^ Department of Thoracic Surgery Zhujiang Hospital Southern Medical University Guangzhou China; ^7^ State Key Laboratory of Respiratory Diseases National Clinical Research Center for Respiratory Disease National Center for Respiratory Medicine Department of Pulmonary and Critical Care Medicine Guangzhou Institute of Respiratory Health The First Affiliated Hospital of Guangzhou Medical University Guangzhou China; ^8^ Department of Pathology Sun Yat‐sen University Cancer Center Guangzhou China; ^9^ Department of Thoracic Surgery Sun Yat‐sen University Cancer Center Guangzhou China

**Keywords:** esophageal squamous cell carcinoma, histone lactylation, immune evasion, lactate, NIPAL1

## Abstract

Metabolic reprogramming is a hallmark of cancer that promotes tumor progression and immune evasion. Here, we identify a NIPAL1‐driven metabolic‐epigenetic circuit in esophageal squamous cell carcinoma (ESCC) that facilitates tumor growth and suppresses antitumor immunity. Mechanistically, NIPAL1 recruits the tyrosine kinase HCK to phosphorylate LDHA at Y10, enhancing glycolysis and lactate production. Lactate accumulation promotes p300‐mediated histone H3K18 lactylation (H3K18la), which transcriptionally activates NIPAL1 expression, establishing a self‐sustaining NIPAL1‐HCK‐p‐LDHA‐lactate‐p300‐H3K18la loop. This axis functions independently of NIPAL1's canonical magnesium transporter activity and promotes immune escape by impairing CD8^+^ T cell function. Pharmacological inhibition of HCK or p300 disrupts this loop and restores antitumor immunity, sensitizing tumors to anti‐PD‐1 therapy. Clinically, expression of NIPAL1, p‐LDHA (Y10), and H3K18la correlates with response to immune checkpoint blockade. Our findings reveal a previously unrecognized NIPAL1‐HCK‐H3K18la signaling loop that integrates tumor metabolism to immune regulation, offering promising targets to improve immunotherapy efficacy in ESCC.

## Graphical abstract

The study identifies a NIPAL1‐driven metabolic‐epigenetic circuit in esophageal squamous cell carcinoma that promotes glycolysis, lactate accumulation, and H3K18 histone lactylation, forming a self‐sustaining loop that suppresses CD8^+^ T cell immunity. Disruption of this NIPAL1‐HCK‐p‐LDHA‐lactate‐p300‐H3K18la axis restores antitumor immunity and sensitizes tumors to anti‐PD‐1 therapy, highlighting potential metabolic‐epigenetic targets for immunotherapy.

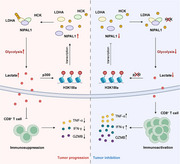

## 1 Introduction

Esophageal squamous cell carcinoma (ESCC), the most common histological subtype of esophageal cancer (EC), remains a highly aggressive malignancy with poor prognosis [1]. Despite recent advances in immunotherapy, particularly immune checkpoint blockade (ICB), clinical responses are limited to a subset of patients [2]. This limited efficacy is largely attributed to immune evasion and the immunosuppressive tumor microenvironment (TME) [3]. Therefore, elucidating the molecular mechanisms that drive tumor progression and immune resistance is critical for identifying novel therapeutic targets and improving outcomes for ESCC patients.

Growing evidence highlights the importance of epigenetic modifications in cancer progression, suggesting their potential as therapeutic agents [4]. Epigenetic dysregulation in tumors typically results from genetic mutations, aberrant enzyme expression, or cofactor imbalances. These disruptions reshape downstream gene expression via histone modifications, chromatin remodeling, and DNA/RNA methylation [5]. Among these, histone modifications profoundly influence chromatin structure and transcriptional regulation [6]. Recent studies have highlighted that lactate, a metabolic byproduct of aerobic glycolysis (the Warburg effect), functions as a key non‐metabolic regulator of epigenetics. Through histone lysine lactylation (Kla), especially at H3K18, lactate modulates transcriptional programs linked to cancer progression [7]. Lactate accumulation in the TME induces extracellular acidification, impairing cytotoxic T lymphocyte (CTL) proliferation and effector function [8, 9]. In addition, lactate restricts glucose uptake in CD8^+^ T cells, thereby suppressing glycolysis and reducing cytotoxic activity [10]. This unbalanced metabolic reprogramming ultimately drives immune escape and unrestrained tumor growth. Although ICB therapy aims to restore CD8^+^ T cell function, its clinical efficacy in ESCC is often limited by the immunosuppressive TME. Given the dual role of lactate in both epigenetic remodeling and immune suppression, elucidating the role of lactate‐induced histone lactylation in ESCC cells and its impact on T cell dysfunction may provide novel insights into overcoming resistance to immunotherapy. However, the upstream regulators that drive lactate accumulation and link metabolic reprogramming to immune evasion in ESCC remain poorly defined.

NIPA‐Like Protein 1 (NIPAL1), also termed magnesium transporter NIPA3, is a membrane protein with multiple predicted transmembrane domains [11]. Emerging evidence suggests that NIPAL1 may contribute to tumorigenesis. Genetic studies have linked NIPAL1 mutations to increased Epstein‐Barr virus susceptibility and impaired antiviral immunity, promoting nasopharyngeal carcinoma development [12]. In oral squamous cell carcinoma (OSCC), NIPAL1 has been implicated in regulating cancer cell proliferation and adhesion [13]. Furthermore, NIPAL1 is transcriptionally activated by Egr1 during macrophage M2 polarization, a process that supports tumor progression [14]. Recent analyses also propose NIPAL1 as a potential prognostic biomarker in pancreatic adenocarcinoma, correlating with immune cell infiltration [15]. Despite these findings, the functional significance of NIPAL1 in modulating metabolic reprogramming and immune evasion in ESCC has not been investigated.

In this study, we identify NIPAL1 as a critical oncogenic driver in ESCC that links metabolic reprogramming to immune evasion. Mechanistically, NIPAL1 recruits HCK to phosphorylate LDHA at residue Y10 and enhances the interaction between HCK and LDHA, thereby boosting glycolytic activity and lactate production. The accumulated lactate promotes p300‐mediated histone H3K18 lactylation (H3K18la), which transcriptionally upregulates NIPAL1 expression, forming a self‐reinforcing NIPAL1‐HCK‐p‐LDHA‐lactate‐p300‐H3K18la signaling axis. This axis suppresses CD8^+^ T cell function and contributes to immunotherapy resistance. Importantly, small‐molecule inhibitors targeting HCK or p300 disrupt the loop and restore antitumor immunity. Clinically, the activity of the NIPAL1‐HCK‐H3K18la axis correlated with ICB responsiveness in ESCC patients. Our findings reveal a novel metabolic‐epigenetic‐immune network that drives ESCC progression and immunotherapy resistance.

## 2 Results

### 2.1 NIPAL1 is Upregulated in ESCC and Promotes Tumor Cell Proliferation

The previous study has identified NIPAL1 as a potential regulatory factor in OSCC patients [13]. Given that both ESCC and OSCC belong to the squamous cell carcinoma subtype and exhibit similar tumor pathologic characteristics, we investigated whether NIPAL1 is also dysregulated in ESCC. Consistent with findings in OSCC, NIPAL1 protein levels were markedly elevated in ESCC tissues compared to matched adjacent non‐tumorous tissues (Figure S1A). To further assess the clinical significance of NIPAL1 expression, we performed immunohistochemistry (IHC) analysis on 52 paired ESCC samples and matched adjacent non‐tumorous tissues. The results demonstrated a significant increase in NIPAL1 expression in tumor tissues (Figure S1B). Importantly, elevated NIPAL1 expression positively correlated with advanced T and N stages (*p* < 0.05; Table S1), and predicted worse patient overall survival (OS) outcomes in ESCC patients (*p* < 0.05; Figure S1C and Table S2). These data suggest a potential oncogenic role for NIPAL1 in ESCC.

To validate the role of NIPAL1 in ESCC progression, we generated two NIPAL1‐knockdown cell lines (KYSE30 and TE1) and one NIPAL1‐overexpressing cell line (KYSE150) (Figure S1D). Knockdown of NIPAL1 significantly inhibited ESCC cell proliferation and colony‐forming ability in vitro (Figure S1E,F), whereas enforced expression of NIPAL1 promoted proliferative capacity (Figure S1G,H). Consistent with the in vitro findings, subcutaneous xenograft experiments in nude mice showed that NIPAL1 knockdown substantially reduced tumor growth and weights in vivo (Figure S1I). Conversely, xenografts derived from NIPAL1‐overexpressing cells exhibited enhanced tumor growth (Figure S1J).

Together, these findings demonstrate that NIPAL1 is highly expressed in ESCC and facilitates ESCC progression by promoting cell proliferation and tumorigenic potential.

### 2.2 NIPAL1 Interacts with LDHA and Modulates Its Activity

**FIGURE 1:**
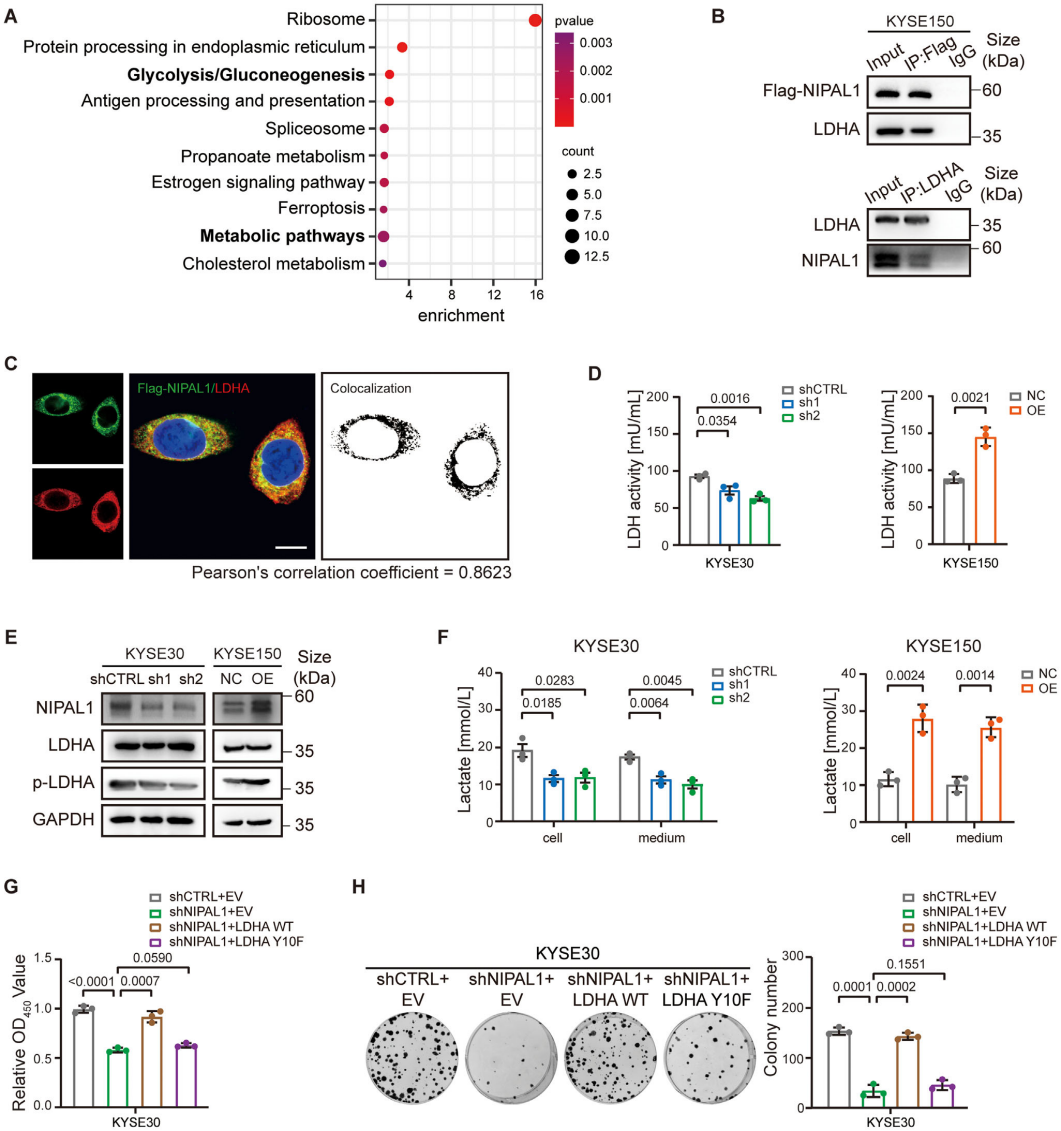
NIPAL1 interacts with LDHA and modulates its activity. (A) Kyoto Encyclopedia of Genes and Genomes (KEGG) pathway enrichment analysis of proteins interacting with NIPAL1, revealing enrichment in metabolic pathways, particularly glycolysis and gluconeogenesis. (B) Western blot of co‐IP assays showing the interaction of NIPAL1 and LDHA in KYSE150 cells. (C) Immunofluorescence (IF) assay showing colocalization of Flag‐NIPAL1 and LDHA in the cytoplasm in KYSE150 cells. Nuclei were stained with DAPI (blue). Scale bar, 10 µm. (D) LDH activity of NIPAL1‐knockdown or NIPAL1‐overexpressing ESCC cells measured by a lactate dehydrogenase activity assay kit. (E) Western blot analysis of total LDHA and p‐LDHA (Y10) levels in NIPAL1‐knockdown or NIPAL1‐overexpressing ESCC cells. (F) Lactate concentrations in cells and culture medium measured by a lactate assay kit. (G) Cell Counting Kit‐8 (CCK‐8) assay showing the viability of KYSE30 cells overexpressing LDHA WT or LDHA Y10F upon NIPAL1 knockdown. (H) Colony formation assay showing the proliferative capacity of KYSE30 cells overexpressing LDHA WT or LDHA Y10F upon NIPAL1 knockdown. Left, representative images. Right, quantification of colony numbers. Data are represented as mean ± S.D. from three independent experiments (D, F, G, H), and the *p* value was determined by a two‐tailed unpaired Student's *t* test (D, F, G, H).

To elucidate the mechanisms by which NIPAL1 promotes ESCC proliferation, we conducted a co‐immunoprecipitation (co‐IP) assay followed by liquid chromatography‐tandem mass spectrometry (LC‐MS/MS) analysis in ESCC cells (Figure S2A). Several distinct interacting proteins (coverage > 0.02) were identified (Table S3), with functional enrichment predominantly with metabolic processes, particularly glycolysis and gluconeogenesis, implicating a potential role of NIPAL1 in modulating glucose metabolism (Figure 1A). Among the candidate proteins identified by LC‐MS/MS, LDHA—an essential enzyme in the Warburg effect—was particularly noteworthy (Figure S2B). We therefore further examined its potential interaction with NIPAL1. Co‐IP assay confirmed the interaction between NIPAL1 and LDHA (Figure 1B). Consistently, the immunofluorescence (IF) assay demonstrated cytoplasmic colocalization of ectopically expressed Flag‐NIPAL1 with endogenous LDHA (Figure 1C).

To determine whether NIPAL1 influences LDHA function, we measured LDHA enzymatic activity following NIPAL1 knockdown or overexpression. NIPAL1 knockdown by two independent shRNAs reduced LDHA activity, whereas its overexpression enhanced enzymatic activity (Figure 1D). Notably, western blot analysis showed that altering NIPAL1 expression did not affect total LDHA protein levels (Figure 1E). Since LDHA phosphorylation at tyrosine 10 (Y10) has been reported to enhance enzymatic activity by stabilizing its tetramer formation, thereby promoting lactate production [16], we hypothesized that NIPAL1 may regulate LDHA activity through this modification. Consistent with this, western blot analysis showed that NIPAL1 knockdown reduced LDHA Y10 phosphorylation, while its overexpression increased phosphorylation at this site (Figure 1E). Furthermore, we observed that knockdown of NIPAL1 significantly decreased both intracellular and extracellular lactate levels, whereas its overexpression led to increased lactate production and release (Figure 1F). Together, these findings suggest that NIPAL1 promotes lactate metabolism by enhancing LDHA enzymatic activity, potentially through modulation of Y10 phosphorylation.

We further investigated whether the Y10 phosphorylation sites on LDHA were responsible for NIPAL1‐mediated ESCC proliferation. Cell Counting Kit‐8 (CCK‐8) and colony formation assays demonstrated that exogenous expression of LDHA wild‐type (WT), but not LDHA Y10F mutant, restored the proliferation effect of NIPAL1‐knockdown cells (Figure 1G,H; Figure S2C). These data highlight the crucial role of LDHA Y10 phosphorylation in mediating the pro‐proliferative effects of NIPAL1.

### 2.3 NIPAL1 Modulates LDHA by Facilitating HCK‐LDHA Interaction

The NIPAL1 protein is composed predominantly of transmembrane and topological regions and lacks intrinsic tyrosine kinase activity. Previous studies have reported that phosphorylation of LDHA at Y10 is catalyzed by upstream kinases such as HER2, FGFR1, and members of the Src family [17]. We screened the LC‐MS/MS proteomic data and identified HCK, a Src family kinase, as a potential NIPAL1‐interacting partner (Figures S2A and S3A).

**FIGURE 2:**
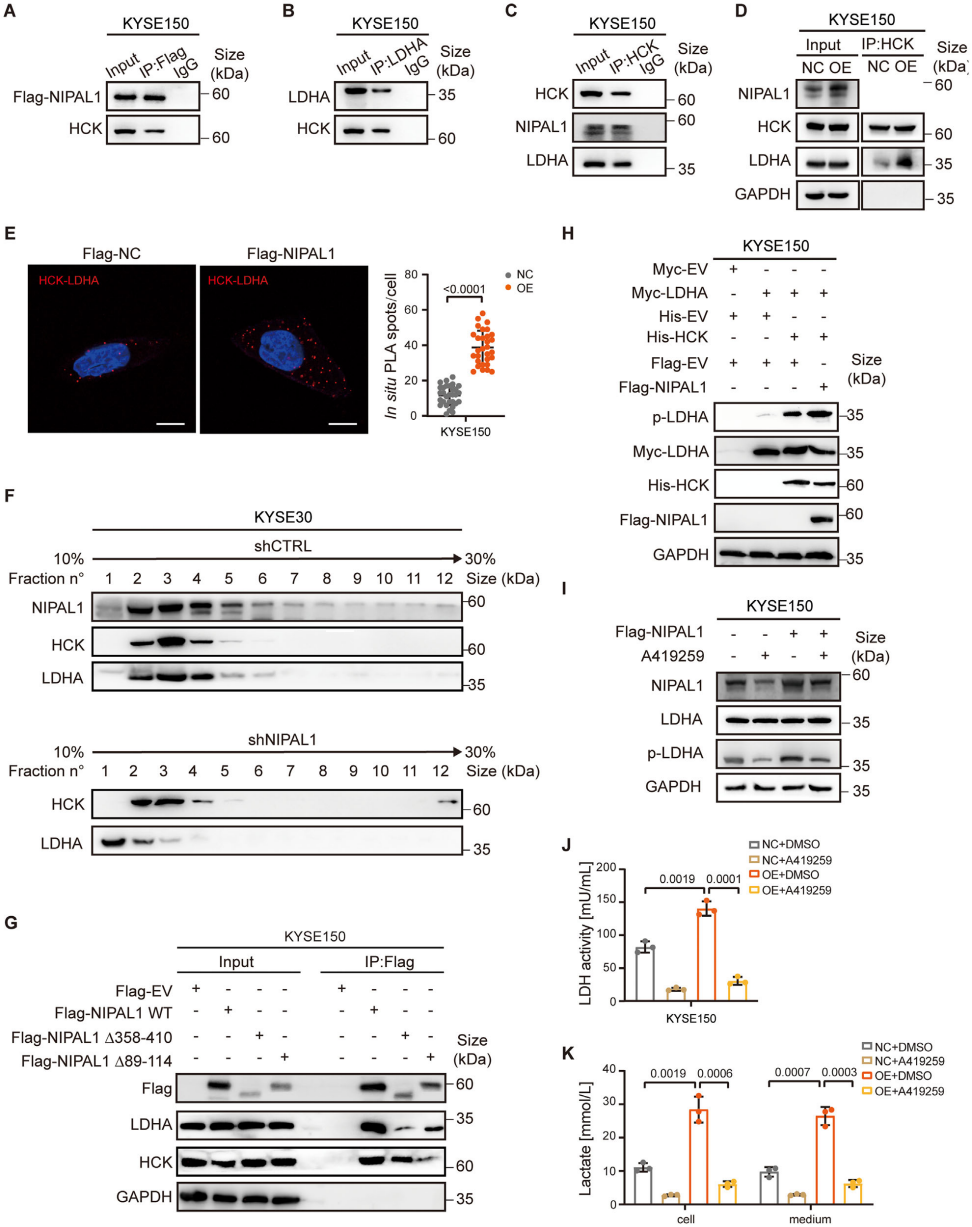
NIPAL1 modulates LDHA by facilitating HCK‐LDHA interaction. (A) Western blot analysis of Flag‐NIPAL1 immunoprecipitates showing the interaction between HCK and NIPAL1 in KYSE150 cells. (B) Western blot analysis of LDHA immunoprecipitates showing the interaction between HCK and LDHA in KYSE150 cells. (C) Western blot analysis of HCK immunoprecipitates showing the binding of both NIPAL1 and LDHA to HCK in KYSE150 cells. (D) Western blot analysis of HCK immunoprecipitates showing increased binding of LDHA to HCK upon NIPAL1 overexpression. (E) Proximity ligation assay (PLA) showing the direct interaction between HCK and LDHA upon NIPAL1 overexpression. Scale bar, 5 µm. (F) Sucrose gradient fraction analysis of the NIPAL1‐HCK‐LDHA complex in control (top) and NIPAL1‐knockdown (bottom) KYSE30 cells. (G) Western blot analysis of LDHA and HCK after co‐IP assay in NIPAL1 WT, Δ358–410, or Δ89–114 transfected KYSE150 cells. (H) Western blot analysis of p‐LDHA in Myc‐LDHA, His‐HCK, and/or Flag‐NIPAL1 transfected KYSE150 cells. (I) Western blot analysis of LDHA and p‐LDHA (Y10) levels in control or NIPAL1‐overexpressing KYSE150 cells treated with A419259. (J) LDH activity in control or NIPAL1‐overexpressing KYSE150 cells treated with A419259. (K) Lactate levels in control or NIPAL1‐overexpressing KYSE150 cells treated with A419259. Data are represented as mean ± S.D. from three independent experiments (J, K), and the *p* value was determined by a two‐tailed unpaired Student's *t* test (E, J, K).

To investigate whether HCK is associated with the NIPAL1/LDHA complex, co‐IP assays were performed. HCK was detected in the immunoprecipitates of both Flag‐NIPAL1 (Figure 2A) and LDHA (Figure 2B). Reciprocal co‐IP assay using anti‐HCK antibody confirmed the association among HCK, NIPAL1, and LDHA (Figure 2C). To assess whether NIPAL1 affects the binding of HCK to LDHA, co‐IP assays were conducted in cells with or without NIPAL1 overexpression. Notably, overexpression of NIPAL1 increased the amount of LDHA co‐immunoprecipitated with HCK (Figure 2D). Moreover, proximity ligation assay (PLA) revealed that NIPAL1 overexpression significantly strengthened the interaction between HCK and LDHA (Figure 2E), suggesting that NIPAL1 facilitates their association. To further validate this, we performed sucrose gradient sedimentation analysis in control and NIPAL1‐knockdown cell lysates. In control cells, NIPAL1, LDHA, and HCK co‐sedimented primarily in fractions 2 to 5, consistent with the presence of a multi‐protein complex. In contrast, NIPAL1 knockdown disrupted this pattern, resulting in reduced LDHA/HCK complexes in fractions 2 and 3, and a shift of LDHA toward fraction 1, indicative of increased monomeric or unbound LDHA (Figure 2F; Figure S3B).

To further elucidate the role of NIPAL1 in mediating the HCK‐LDHA interaction, molecular docking simulations were performed to predict the structural organization and binding patterns within the complex. Docking analysis predicted that HCK and LDHA can form a complex through multiple interaction interfaces. Notably, HCK and LDHA were predicted to bind to distinct regions on NIPAL1 (Figure S3C), suggesting that NIPAL1 may influence the interaction mode between HCK and LDHA. To validate this prediction, we generated two NIPAL1 truncation constructs, selectively deleting the predicted LDHA‐binding region (Δ358–410) or the HCK‐binding region (Δ89–114) (Figure S3D). Co‐IP assays following transfection with NIPAL1 WT or truncation mutants revealed that the deletion of residues 358–410 partially impaired the interaction between NIPAL1 and LDHA, whereas deletion of residues 89–114 partially attenuated the interaction between NIPAL1 and HCK (Figure 2G). These results support a model in which HCK and LDHA can each directly bind NIPAL1, while also engaging NIPAL1 indirectly through their mutual interaction. In addition, formation of the ternary complex leads to alterations in the predicted interaction interfaces between LDHA and HCK (Figure S3C), suggesting that NIPAL1‐mediated recruitment of HCK to LDHA induces conformational rearrangements that may facilitate exposure of functionally active sites.

To assess the effect of HCK kinase activity on NIPAL1‐mediated LDHA phosphorylation, LDHA was co‐expressed with HCK. Co‐expression of HCK led to a substantial increase in LDHA tyrosine phosphorylation (Figure 2H), which was further enhanced upon co‐transfection with NIPAL1, indicating that HCK acts as a physiological modifier of LDHA and that NIPAL1 can potentiate this process. Moreover, overexpression of NIPAL1 enhanced LDHA phosphorylation at Y10, which was attenuated in a dose‐dependent manner by the HCK inhibitor A419259 (Figure S3E). Furthermore, NIPAL1‐induced increases in LDHA phosphorylation, enzymatic activity, lactate production, and cell proliferation were abolished by HCK inhibition (Figure 2I–K; Figure S3F,G). Collectively, these data suggest that NIPAL1 promotes LDHA activation and lactate metabolism through an HCK‐dependent mechanism.

Given that NIPAL1 belongs to the family of magnesium transporters, but its ion‐flux activity remains uncharacterized, we investigated whether NIPAL1 modulates lactate production through magnesium flux. ESCC cells with either NIPAL1 knockdown or overexpression were pretreated with 2 mM MgCl_2_. Co‐IP assays showed that extracellular Mg^2^^+^ supplementation did not further strengthen the interaction between LDHA and Flag‐tagged NIPAL1 (Figure S4A). Moreover, NIPAL1 knockdown reduced the amount of LDHA co‐immunoprecipitated with HCK, while MgCl_2_ exposure failed to restore this reduction (Figure S4B). Consistently, extracellular Mg^2^^+^ uptake did not affect NIPAL1‐mediated lactate production (Figure S4C,D). These findings demonstrate that the regulatory effect of NIPAL1 on lactate metabolism is independent of any magnesium‐transport function.

### 2.4 NIPAL1 Expression is Upregulated by Tumor‐Derived Lactate through Histone Lactylation

**FIGURE 3:**
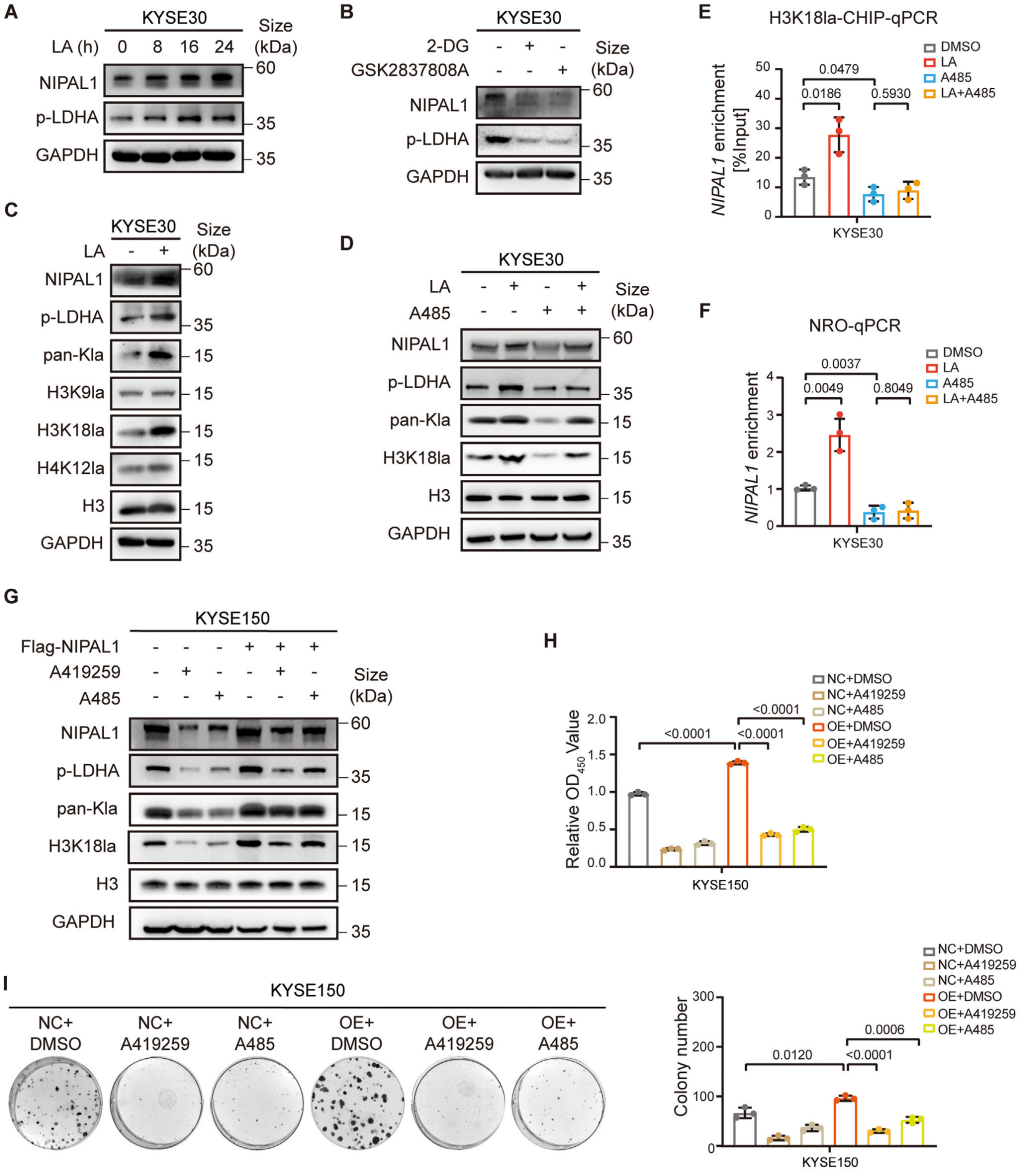
NIPAL1 expression is upregulated by tumor‐derived lactate through histone lactylation. (A) Western blot analysis of NIPAL1 and p‐LDHA (Y10) levels in KYSE30 cells treated with 25 mM lactic acid (LA) at the indicated time. (B) Western blot analysis of NIPAL1 and p‐LDHA (Y10) levels in KYSE30 cells treated with 2‐deoxyglucose (2‐DG) or GSK2837808A. (C) Western blot analysis of the indicated protein levels in KYSE30 cells treated with 25 mM LA. (D) Western blot analysis of the indicated protein levels in KYSE30 cells treated with LA and/or A485. (E) Chromatin immunoprecipitation (ChIP) assay showing enrichment of H3K18la at the *NIPAL1* promoter in KYSE30 cells treated with LA and/or A485. (F) Nuclear run on (NRO) assay showing transcriptional regulation of *NIPAL1* in KYSE30 cells treated with LA and/or A485. (G) Western blot analysis of the indicated proteins in NIPAL1‐overexpressing KYSE150 cells treated with A419259 or A485. (H) CCK‐8 assay showing the viability of NIPAL1‐overexpressing KYSE150 cells treated with A419259 or A485. (I) Colony formation assay showing the proliferative capacity of NIPAL1‐overexpressing KYSE150 cells treated with A419259 or A485. Data are represented as mean ± S.D. from three independent experiments (E, F, H, I), and the *p* value was determined by a two‐tailed unpaired Student's *t* test (E, F, H, I).

Our results indicate that NIPAL1 is highly expressed in ESCC, but the mechanism remains undefined. To address this, we investigated whether lactate, the end product of glucose metabolism, drives NIPAL1 expression. Treatment with lactic acid (LA) led to a time‐dependent increase in NIPAL1 protein and LDHA Y10 phosphorylation in ESCC cells (Figure 3A). In contrast, inhibition of glycolysis with 2‐deoxyglucose (2‐DG) or LDHA activity with GSK2837808A diminished both NIPAL1 expression and LDHA phosphorylation (Figure 3B).

Since histone lactylation has been reported to activate gene transcription [18], we evaluated its role in NIPAL1 expression. LA exposure elevated *NIPAL1* mRNA and protein, as well as global lysine lactylation (pan‐Kla) and H3K18la, whereas levels of H3K9la and H4K12la were unaffected (Figure 3C; Figure S5A). Acetyltransferase p300 is established as a key “writer” for histone lactylation [7]. In this study, we observed that treatment with the p300 inhibitor A485 reduced basal pan‐Kla, H3K18la, and NIPAL1, and prevented their upregulation induced by LA (Figure 3D). Chromatin immunoprecipitation (ChIP) using an anti‐H3K18la antibody followed by qPCR revealed significant enrichment of H3K18la at the *NIPAL1* promoter following LA treatment, which was abolished by A485 (Figure 3E). Consistently, nuclear run‐on (NRO) assay showed that LA increased transcriptional activity at the *NIPAL1* promoter, and this effect was reversed by p300 inhibition (Figure 3F). Further anti‐Flag immunoprecipitation revealed that NIPAL1 itself is not directly lactylated (Figure S5B). Given that lactate production is amplified by NIPAL1‐dependent LDHA activation, we hypothesized a positive feedback loop linking metabolic reprogramming and histone lactylation. Indeed, inhibition of either HCK or p300 decreased NIPAL1 expression, H3K18la levels, and LDHA phosphorylation (Figure 3G), and impaired NIPAL1‐driven cell proliferation (Figure 3H,I). Notably, treatment with either the HCK inhibitor A419259 or the p300 inhibitor A485 reduced NIPAL1 protein levels even under ectopic overexpression (Figures 2I and 3G), supporting a self‐sustaining feedback loop in which HCK‐mediated LDHA phosphorylation promotes lactate accumulation that drives p300‐dependent H3K18la to maintain NIPAL1 transcription. Disruption of HCK or p300 breaks this circuit, highlighting the interdependence of metabolic and epigenetic regulation in sustaining NIPAL1 levels.

Collectively, these data support a positive feedback regulatory loop in which lactate‐p300‐H3K18la and HCK‐mediated LDHA activation cooperate to maintain NIPAL1 expression and promote tumor progression.

### 2.5 The NIPAL1‐HCK‐H3K18la Loop Modulates Glycolysis in ESCC

**FIGURE 4:**
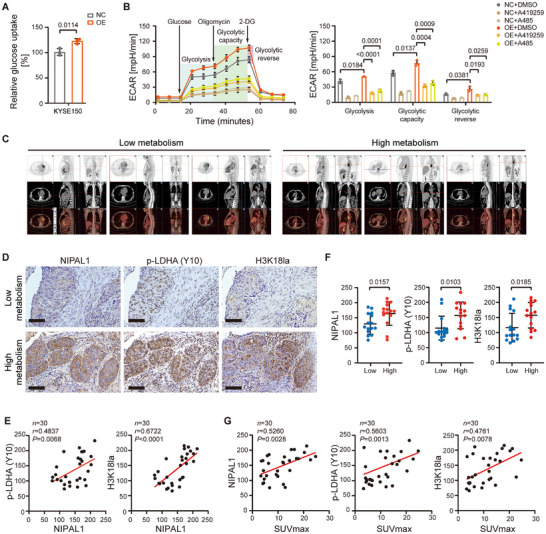
The NIPAL1‐HCK‐H3K18la loop modulates glycolysis in ESCC. (A) Relative glucose uptake in control or NIPAL1‐overexpressing KYSE150 cells. (B) Glycolysis flux assessed by ECAR using the Seahorse analyzer in NIPAL1‐overexpressing KYSE150 cells treated with A419259 or A485. (C) Representative PET‐CT images of ESCC patients classified into low‐ and high‐glucose metabolism groups. (D) Representative IHC staining images of NIPAL1, p‐LDHA (Y10), and H3K181la in two ESCC tissues from low‐metabolism (top row) or high‐metabolism (bottom row) groups. Scale bar, 100 µm. (E) Correlation analysis of the expression levels of p‐LDHA (Y10) and H3K18la with NIPAL1 expression in ESCCs (*n* = 30). (F) Relative expression levels of NIPAL1 (left), p‐LDHA (Y10) (middle), and H3K18la (right) in low‐ and high‐metabolism groups (*n* = 30). (G) Correlation analysis of the relative expression levels of NIPAL1 (left), p‐LDHA (Y10) (middle), and H3K18la (right) with tumor SUVmax in ESCC patients (*n* = 30). Data are represented as mean ± S.D. from three independent experiments (A, B), and the *p* value was determined by a two‐tailed unpaired Student's *t* test (A, B, F) or Pearson correlation analysis (E, G).

Given the central role of LDHA in glycolysis, we hypothesized that NIPAL1 binding could influence glucose metabolism and lactate generation. To test this, we quantified glucose uptake and lactate secretion in ESCC cells. Compared with controls, NIPAL1‐overexpressing cells exhibited a marked rise in both glucose import and lactate release, supporting a pro‐glycolytic function for NIPAL1 (Figures 1F and 4A). Measurement of extracellular acidification rate (ECAR) using the Seahorse Analyzer demonstrated that NIPAL1 overexpression enhanced glycolytic flux in ESCC cells, an effect that was abolished by either HCK inhibitor or p300 inhibitor (Figure 4B). These findings suggest that NIPAL1 promotes glycolysis through the HCK‐p‐LDHA‐lactate‐p300‐H3K18la signaling axis.

18 fluorodeoxyglucose positron emission‐computed tomography (^18^F‐FDG PET/CT) provides a noninvasive means to visualize tumor biology and qualify in vivo tumor glucose metabolism, with the maximum standardized uptake value (SUV_max_) serving as the quantitative indicator of glucose uptake [19]. 30 ESCC patients were divided into high‐metabolism and low‐metabolism groups based on their ^1^ ^8^F‐FDG PET/CT SUV_max_ values (Figure 4C). IHC analysis revealed that NIPAL1 expression positively correlated with LDHA Y10 phosphorylation and H3K18la levels (Figure 4D,E). Moreover, tumor tissues from patients with high metabolic activity exhibited significantly elevated NIPAL1, p‐LDHA (Y10), and H3K18la compared to those with low metabolic activity (Figure 4D,F). Statistical analysis demonstrated significant positive associations among NIPAL1, p‐LDHA (Y10), H3K18la, and SUV_max_ values (Figure 4G). These clinical data support a model in which NIPAL1‐mediated reprogramming of glucose metabolism contributes to ESCC progression.

### 2.6 NIPAL1 Modulates CD8^+^ T Cell Activity through Increased Lactate Secretion to the Tumor Microenvironment

Our data indicate that NIPAL1 enhances both lactate production and secretion in ESCC cells (Figure 1F). Lactate is typically exported from tumor cells into the tumor microenvironment via monocarboxylate transporters (MCTs), where it modulates the function and behavior of various surrounding cell populations [20]. Given that NIPAL1 belongs to the solute carrier family and contains multiple predicted transmembrane domains, we sought to determine whether it contributes to lactate secretion via a transporter‐dependent mechanism. To this end, we performed subcellular fractionation of ESCC cells to isolate plasma membrane and intracellular organelle membrane components. Western blot analysis revealed that NIPAL1 was not enriched in the plasma membrane fraction, but predominantly associated with the mitochondrial, Golgi, and endoplasmic reticulum membrane fractions (Figure S6A). This subcellular distribution pattern was further supported by IF staining, which revealed that NIPAL1 primarily resides on intracellular organelle membranes rather than the plasma membrane (Figure S6B). These findings suggest that NIPAL1 does not directly mediate lactate export across the plasma membrane but instead regulates lactate metabolism through transporter‐independent mechanisms, possibly involving intracellular signaling or metabolic modulation.

**FIGURE 5:**
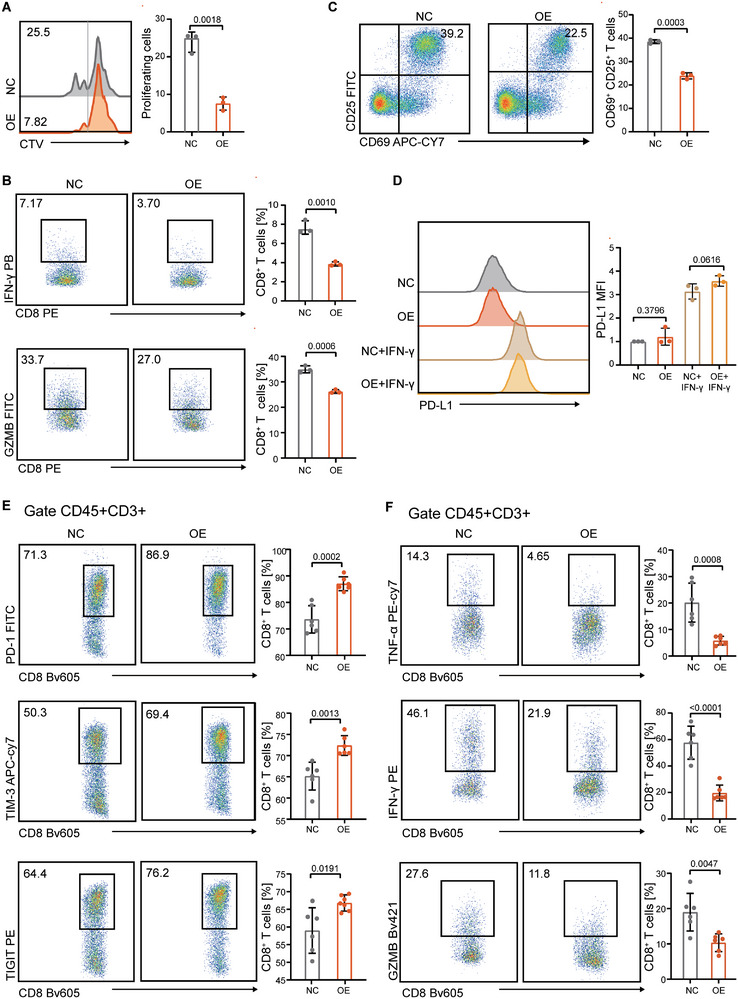
NIPAL1 modulates CD8^+^ T cell activity through increased lactate secretion to the tumor microenvironment. (A) Flow cytometric analysis of CD8^+^ T cell proliferation after co‐culture with control or NIPAL1‐overexpressing KYSE150 cells. (B) Representative contour plots showing expression of the indicated cytokines in CD8^+^ T cells after co‐culture with control or NIPAL1‐overexpressing KYSE150 cells. (C) Flow cytometric analysis of T cell activation by measuring surface expression levels of CD69 and CD25. (D) MFI of PD‐L1 in control or NIPAL1‐overexpressing KYSE150 cells after IFN‐γ treatment. (E) Flow cytometric analysis of tumor‐infiltrating PD‐1^+^CD8^+^ T cells, TIM‐3^+^CD8^+^ T cells, and TIGIT^+^CD8^+^ T cells in tumors derived from subcutaneous injection of control or NIPAL1‐overexpressing MC38 cells (*n* = 6). (F) Flow cytometric analysis of TNF‐α, IFN‐γ, and GZMB production of tumor‐infiltrating CD8^+^ T cells in tumors derived from subcutaneous injection of control or NIPAL1‐overexpressing MC38 cells (*n* = 6). Data are represented as mean ± S.D. from three independent experiments (A–D), and the *p* value was determined by a two‐tailed unpaired Student's *t* test (A–F).

Previous studies have reported that elevated extracellular lactate, driven by tumor‐intrinsic signaling, can impair the cytotoxic function of CD8^+^ T cells [8, 21]. To explore whether NIPAL1‐mediated lactate accumulation exerts similar immunomodulatory effects in ESCC, we first assessed T cell proliferation using the CellTrace Violet (CTV) incorporation assay. Co‐culture with NIPAL1‐overexpressing ESCC cells significantly inhibited T cell proliferation (Figure 5A). Furthermore, CD8^+^ T cells exhibited markedly reduced cytokine production following incubation with NIPAL1‐overexpressing cells (Figure 5B). Given that T cell activation is characterized by the upregulation of early surface markers CD69 and CD25, we next investigated whether NIPAL1 regulated T cell activation. Flow cytometry revealed a significant reduction in the proportion of CD25^+^CD69^+^ T cells in the NIPAL1‐overexpression group (Figure 5C), suggesting that NIPAL1 suppresses T cell activation, potentially contributing to reduced proliferation and cytokine secretion. Previous studies have demonstrated that lactate suppresses T cell function through multiple mechanisms—directly impairs T cell activity and simultaneously upregulates the expression of the co‐inhibitory molecule PD‐L1 in tumor cells [22, 23]. We therefore evaluated PD‐L1 expression under IFN‐γ stimulation. However, no significant difference in PD‐L1 levels was observed between control and NIPAL1‐overexpressing cells (Figure 5D), indicating that NIPAL1 does not regulate PD‐L1 under these conditions.

The MC38 tumor model is widely used for the preclinical evaluation of immunotherapeutic strategies. To further evaluate the in vivo immunomodulatory role of NIPAL1, we established subcutaneous xenografts using MC38 cells in C57BL/6J mice. Flow cytometric analysis showed that the expression of exhaustion markers PD‐1, TIM‐3, and TIGIT on CD8^+^ T cells was significantly increased in tumors with high NIPAL1 expression (Figure 5E). Moreover, this was accompanied by a marked reduction in cytokine‐producing CD8^+^ T cells in the NIPAL1‐overexpression group (Figure 5F).

To determine whether CD8^+^ T cell suppression is mediated by soluble factors, conditioned medium (CM) was collected from control or NIPAL1‐overexpressing tumor cells and applied to CD8^+^ T cells in the absence of direct cell‐cell contact. CM from NIPAL1‐overexpressing cells significantly inhibited CD8^+^ T cells' proliferation. Notably, this inhibitory effect was abolished when tumor cells were pretreated with the LDHA inhibitor GSK2837808A, the upstream HCK inhibitor A419259, or the p300 inhibitor A485 (Figure S7). These findings indicate that NIPAL1‐induced suppression of CD8^+^ T cell function is mediated by tumor‐derived soluble factors and is dependent on the HCK‐lactate‐p300 signaling axis.

Collectively, these results support the view that NIPAL1 impairs CD8^+^ T cell‐mediated antitumor immunity, likely through lactate‐driven immunosuppression, contributing to immune evasion in the tumor microenvironment.

### 2.7 Targeting NIPAL1‐HCK‐H3K18la Loop Sensitizes Tumors to Checkpoint Therapy

**FIGURE 6:**
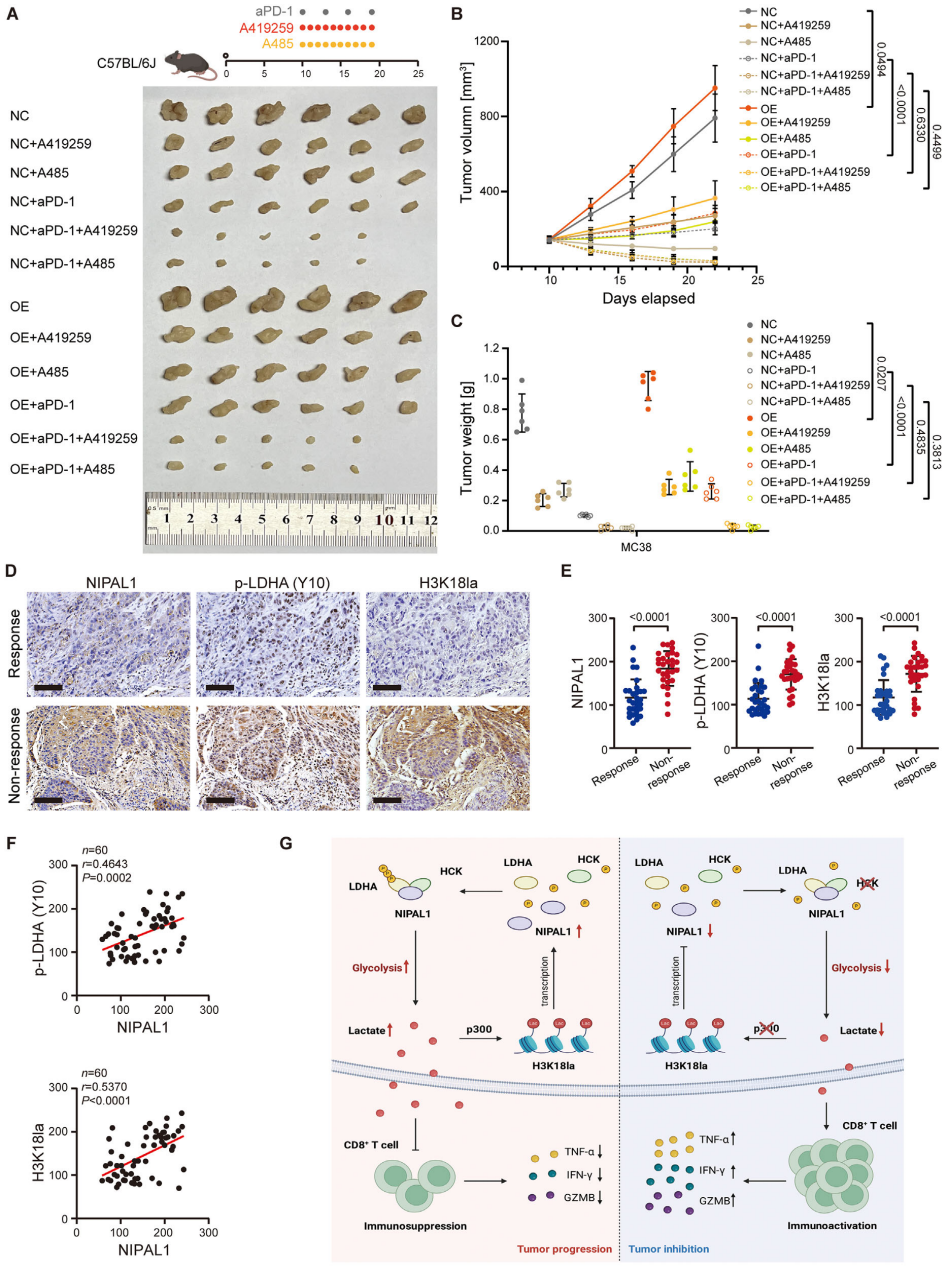
Targeting NIPAL1‐HCK‐H3K18la loop sensitizes cells to checkpoint therapy. (A–C) Subcutaneous xenograft tumor model showing the impact of A419259, A485, and/or anti‐PD‐1 treatment in C57BL/6J mice injected with NIPAL1‐overexpressing MC38 cells (*n* = 6). (A) Subcutaneous tumor images. (B) Quantification of subcutaneous tumor volume. (C) Quantification of subcutaneous tumor weights. (D) Representative IHC staining images of NIPAL1, p‐LDHA (Y10), and H3K181la in two ESCC tissues from response (top row) or non‐response (bottom row) groups. Scale bar, 100 µm. (E) Relative expression levels of NIPAL1 (left), p‐LDHA (Y10) (middle), and H3K18la (right) with immunotherapy response in ESCCs (*n* = 60). (F) Correlation analysis of the expression levels of p‐LDHA (Y10) (top) and H3K18la (bottom) with NIPAL1 expression in ESCCs (*n* = 60). (G) A proposed model illustrating that the NIPAL1‐mediated metabolic‐epigenetic positive feedback loop (NIPAL1/HCK/H3K18la) not only drives ESCC progression through enhanced glycolysis but also contributes to immunotherapy resistance by suppressing CD8^+^ T cell function through immune metabolic reprogramming. The *p* value was determined by a two‐tailed unpaired Student's *t* test (B, C, E) or Pearson correlation analysis (F).

Given the critical role of lactate in sustaining T‐cell exhaustion, therapeutic strategies targeting lactate metabolism, such as LDHA inhibitors [24] or MCT4 inhibitors [25], may synergize with immune checkpoint inhibitors (ICIs) by reprogramming dysfunctional T cells and restoring their antitumor activity. Since our earlier findings demonstrated that the NIPAL1‐HCK‐H3K18la loop modulates lactate secretion, we next investigated whether disrupting this regulatory circuit could improve immunotherapeutic outcomes. Indeed, treatment of mice bearing NIPAL1‐overexpressing MC38 tumors with either an HCK inhibitor or a p300 inhibitor in combination with an anti‐PD‐1 antibody significantly suppressed tumor growth compared with monotherapies or control treatments (Figure 6A–C).

To assess the clinical relevance of these findings, we examined the expression levels of NIPAL1, p‐LDHA (Y10), and H3K18la by IHC in pre‐treatment biopsy specimens from a cohort of 60 ESCC patients who subsequently received neoadjuvant immunotherapy‐based treatment. Notably, patients with poor response to ICIs treatment exhibited significantly higher levels of NIPAL1, p‐LDHA (Y10), and H3K18la than those with a favorable response (Figure 6D,E). Consistent with our previous results, NIPAL1 expression was positively correlated with both p‐LDHA (Y10) and H3K18la levels (Figure 6F).

Collectively, these data suggest that the NIPAL1‐HCK‐H3K18la signaling axis drives resistance to ICB in ESCC. Targeting this positive feedback loop may enhance therapeutic efficacy in resistant tumors.

## 3 Discussion

Metabolic reprogramming is a hallmark of cancer, playing a pivotal role in tumor progression and shaping the immunosuppressive TME [26, 27]. In this study, we uncovered a previously unrecognized metabolic‐epigenetic axis driven by NIPAL1 that promotes tumor progression and immune evasion in ESCC. Mechanistically, NIPAL1 enhances glycolytic flux and lactate production by recruiting the tyrosine kinase HCK, which phosphorylates LDHA at tyrosine 10, thereby augmenting its enzymatic activity. Notably, intracellular lactate accumulation promotes p300‐mediated H3K18la, which in turn transcriptionally activates NIPAL1 expression, establishing a self‐sustaining NIPAL1‐HCK‐p‐LDHA‐lactate‐p300‐H3K18la feedback loop. This epigenetic‐metabolic circuit amplifies oncogenic signaling and tumor‐supportive epigenetic modification, ultimately contributing to the poor clinical prognosis in ESCC patients.

A key mechanistic insight from this study is the identification of an NIPAL1‐HCK‐p‐LDHA signaling axis that regulates glycolysis via tyrosine phosphorylation. LDHA catalyzes the final step of glycolysis by converting pyruvate to lactate and plays a central role in tumor‐associated metabolic reprogramming [28]. Rather than altering LDHA expression, NIPAL1 enhances its enzymatic activity by recruiting the Src family tyrosine kinase HCK, which phosphorylates LDHA at Y10, a modification known to promote LDHA tetramer formation and augment its catalytic capacity [16]. Although NIPAL1 belongs to the magnesium transporter‐like NIPA family [11], our results suggest that its regulatory role in lactate production is independent of ion transport activity. Instead, NIPAL1 predominantly localizes to organelle membranes and modulates the spatial conformation of HCK and LDHA, thereby enhancing their interaction. This previously unrecognized regulatory role of NIPAL1 broadens the current understanding of transmembrane proteins, extending their roles in cancer metabolism. Given that HCK is a pharmacologically targetable Src‐family kinase [29], the NIPAL1‐HCK‐p‐LDHA axis may represent a promising target to disrupt glycolysis and downstream lactate‐driven oncogenic signaling in ESCC.

Lactate has recently emerged as a key metabolic intermediate linking cellular metabolism to epigenetic regulation through a novel post‐translational modification known as histone lactylation. This lactate‐derived modification has been shown to modulate gene transcription and cell fate decisions [30]. Accumulation of lactate, a result of metabolic reprogramming driven by oncogenic signaling, can induce significant changes in histone lactylation patterns, including modifications at H3K9, H3K18, H4K5, and H4K12 [23, 31, 32], which have been implicated in tumor progression. While NIPAL1 has been reported to be dysregulated in multiple cancers, its involvement in lactate‐induced histone modifications has not been addressed. Our findings demonstrated that NIPAL1 promotes not only lactate production but also epigenetic remodeling via H3K18la, thereby establishing a self‐sustaining metabolic‐epigenetic loop. This mechanism aligns with the NSUN2/YBX1/m^5^C‐ENO1‐lactate‐H3K18la axis previously reported in colorectal cancer, where H3K18la upregulation reinforces NSUN2 transcription to drive tumor progression [33]. Our study extends this concept to ESCC and identifies NIPAL1 as a novel mediator of lactate‐driven H3K18la feedback regulation.

In addition to promoting epigenetic remodeling, lactate also exerts profound immunosuppressive effects within the TME. Lactate shuttling among diverse cell populations within the TME, a phenomenon known as metabolic symbiosis [34], can inhibit immune cell differentiation, impair effector function, and promote T cell exhaustion [35]. Previous studies have shown that LDHA‐deficient melanoma cells exhibit enhanced infiltration of CD8^+^ T and NK cells [36], and that lactate accumulation in KRAS‐mutant colorectal cancer cells suppresses anti‐PD‐1 therapy efficacy by impairing NF‐κB signaling [37]. In a humanized mouse model of non‐small cell lung cancer, LDH inhibition combined with PD‐1 blockade enhanced CD8^+^ T cell infiltration and suppressed tumor growth, sensitizing tumors to immunotherapy [24]. Additionally, blocking MCTs that mediate lactate shuttling can disrupt metabolic symbiosis, enhance tumor‐reactive CD8^+^ T cell function, and improve immunotherapeutic responses in colon, melanoma, and breast cancers [38]. Consistent with these observations, our data indicate that NIPAL1‐driven lactate metabolism suppresses CD8^+^ T cell function through tumor‐derived soluble mediators, highlighting a contact‐independent immunosuppressive mechanism. This effect is largely dependent on the HCK‐lactate‐p300 signaling axis, linking metabolic reprogramming to immune dysfunction in the TME. Given that extracellular acidification can impair CD8^+^ T cell cytotoxicity [39, 40], all CM used for T cell stimulation were supplemented with 25 mM HEPES to maintain a stable physiological pH throughout the co‐culture period, thereby minimizing potential confounding effects of lactate‐induced acidification. While adjusting medium pH with HCl to match lactate‐rich conditioned medium would represent a more direct approach for distinguishing the effects of lactate from low pH alone [41], our HEPES‐buffering strategy limits pH fluctuations while preserving the full repertoire of tumor‐derived soluble factors, indicating that the immunosuppressive effect is unlikely to be solely attributable to nonspecific acidification. Although our findings support an important role for lactate, future work will involve quantitative measurement of lactate in CM and correlation analyses to clarify its relative contribution among tumor‐derived soluble factors. We next assessed the therapeutic relevance of this metabolic‐epigenetic axis. Notably, pharmacological inhibition of either HCK or the epigenetic writer p300 disrupted the lactate‐driven feedback loop and restored anti‐tumor immunity, significantly enhancing the efficacy of anti‐PD‐1 therapy in vivo. Collectively, these results support a functional role for lactate downstream of NIPAL1 in shaping the immunosuppressive TME.

These findings support the existence of an immunoregulatory axis, NIPAL1‐HCK‐H3K18la, that integrates metabolic reprogramming with epigenetic modification to drive immune suppression. This axis not only exacerbates tumor aggressiveness but also undermines immune surveillance, contributing to resistance against ICIs. As the clinical benefits of ICIs in ESCC remain limited due to the immunosuppressive nature of the TME and the complexity of resistance mechanisms, combination strategies targeting multiple layers of tumor biology are urgently required. Our results demonstrate that NIPAL1‐mediated metabolic reprogramming significantly impairs CD8^+^ T cell function and promotes immune escape. Notably, both HCK and p300 inhibitors showed potent effects in disrupting the NIPAL1 feedback loop and enhancing PD‐1 blockade efficacy, underscoring their therapeutic potential. Clinically, the expression levels of NIPAL1, p‐LDHA, and H3K18la positively correlated with response to ICB, indicating their promise as predictive biomarkers to stratify patients for combination therapies involving metabolic or epigenetic inhibitors and ICB.

In conclusion, our study reveals a previously uncharacterized metabolic‐epigenetic‐immune crosstalk driven by NIPAL1 in ESCC (Figure 6G). We identify the NIPAL1‐HCK‐p‐LDHA‐lactate‐p300‐H3K18la cascade as a self‐reinforcing signaling loop that promotes tumor growth and immune evasion. Targeting this axis not only reprograms tumor metabolism but also restores CD8^+^ T cell function and sensitizes tumors to ICB. These findings provide a strong rationale for targeting metabolic‐epigenetic pathways in ESCC, particularly the NIPAL1‐driven signaling cascade, to enhance responsiveness to immunotherapy and improve patient outcomes.

## 4 Experimental Section

### 4.1 Human Specimen

For western blot analysis, 12 primary ESCC tissues and matched normal tissues were collected at Guangdong Provincial People's Hospital (GDPH) in 2021. For IHC analysis in Figure S1, 52 treatment‐naïve ESCC samples were collected at GDPH between 2018 and 2021, among which 30 cases with complete PET/CT reports were also used for IHC analysis in Figure 4. For IHC analysis in Figure 6, samples from 60 patients receiving immunotherapy were prospectively collected. The ESCC grade and stage were evaluated by the criteria of the WHO classification (2016) and the eighth edition of the TNM classification of the UICC (2017). The Response Evaluation Criteria in Solid Tumors (RECIST) version 1.1 was applied to determine the time to progression and tumor response for each patient. The cutoff date for the last follow‐up was June 2023.

### 4.2 Cell Culture

The KYSE30 (RRID: CVCL_1351), KYSE150 (RRID: CVCL_1348), TE1 (RRID: CVCL_1759), 293T (RRID: CVCL_0045), and MC38 (RRID: CVCL_B288) cell lines were purchased from the American Type Culture Collection in 2022. All cell lines were confirmed to be free from cross‐contamination and tested negative for mycoplasma contamination before use. All cells were cultured for less than 2 months at 37°C with 5% CO_2_ in DMEM (Invitrogen, California, USA) supplemented with 10% fetal bovine serum (FBS) (PAN, Aidenbach, Germany) and 1% antibiotics.

### 4.3 Animal Models

The 4‐week‐old female BALB/c (RRID: MGI:2161072) nude mice or 6‐week‐old female C57BL/6J (RRID: IMSR_JAX:000664) were procured from the Guangdong Provincial Laboratory Animal Center (Foshan, China), and housed under specific pathogen‐free conditions of the Sun Yat‐sen University Cancer Center (Guangzhou, China). Female mice were used to reduce variability in tumor growth and immune response associated with sex differences.

For the KYSE30 and KYSE150 models, 5 × 10^6^/100 µL cells were subcutaneously injected into the right flank of BALB/c nude mice (*n* = 6/group). After 3 weeks, the mice were euthanized, and the subcutaneous tumors were weighed.

For the MC38 models, 5 × 10^5^/100 µL cells were subcutaneously injected into the right flank of C57BL/6J mice (*n* = 6/group). To evaluate the combinatorial effect of inhibitor and immunotherapy on tumor growth, mice received anti‐PD‐1 antibody (10 mg/Kg/BW) alone or in combination with A419259 (30 mg/Kg/QD) or A485 (20 mg/Kg/QD) after tumor implantation for 10 days. Tumor size was routinely monitored every 3 days. Tumor volumes were calculated by the following formula: tumor volume = 0.5 × length × width^2^. After 10 days, the mice were euthanized, and the subcutaneous tumors were weighed.

### 4.4 Western Blot

Proteins were isolated by IP lysis buffer (50 mM Tris‐HCl, pH 7.4, 150 mM NaCl, 1 mM EDTA, 0.5% NP‐40, and 10% Glycerol) supplemented with protease inhibitor Cocktail (CWBIO, Beijing, China) and quantified by using the BCA protein assay kit (GLPBIO, Montclair, USA). Protein lysates were separated in 10% SDS‐PAGE and transferred to PVDF membranes (Roche, Basel, Switzerland). After blocking with 5% non‐fat milk for 1 h, the membranes were incubated with primary antibodies at 4°C overnight and corresponding secondary antibodies at room temperature for 1 h. The protein signals were detected by Chemistar High‐sig ECL Western blot Substrate (Tanon, Shanghai, China). Details of the antibodies used are provided in Table S4.

### 4.5 IHC Staining

The paraffin tissues were hydrated and incubated with 0.3% hydrogen peroxide for 15 min at room temperature, followed by high‐pressure antigen retrieval in EDTA buffer (PH 8.0). The sections were blocked in 1:50 goat serum (CWBio, Beijing, China) for 1 h at room temperature and incubated with primary antibodies at 4°C overnight. Universal secondary antibody (ZSGB‐Bio, Beijing, China) was incubated on the slide for 1 h at room temperature. Immunoreactivity was detected using a DAB reagent kit (ZSGB‐Bio, Beijing, China). Images were captured using a Jiangfeng scanner (KF‐PRO‐020, China). The NIPAL1 staining was assessed independently by two pathologists. For each sample, a histoscore (H score) ranging from 0 to 300 was calculated as follows: H score = (1 × % weakly stained cells) + (2 × % moderately stained cells) + (3 × % strongly stained cells). An optimal cut‐off value was defined according to the median value of the patient cohort. Details of the antibodies used are provided in Table S4.

### 4.6 Plasmid Construction and Transfection

The NIPAL1 overexpression plasmid, Flag‐NIPAL1, Flag‐NIPAL1 Δ358‐410, Flag‐NIPAL1 Δ89‐114, Myc‐LDHA WT, Myc‐LDHA Y10F, and His‐HCK plasmids were obtained from GeneCreate Biotech (Wuhan, China). The plasmid psi‐LVRU6GP was used for constructing the NIPAL1 shRNAs obtained from GeneCopoeia (MD, USA). The target sequences for constructing shRNAs are provided in Table S5.

The purified transfer plasmid, along with envelope plasmid pMD2.G (RRID: Addgene_12259) and packaging plasmid psPAX2 (GeneCopoeia, MD, USA, RRID: Addgene_12260), was transfected into 293T cells using jetPRIME reagent (Polyplus, Strasbourg, France). The viral supernatant was collected at 48 h post‐transfection. Stable transduced cells were selected using puromycin.

### 4.7 Reverse Transcription‐Quantitative Polymerase Chain Reaction (RT‐qPCR)

Total RNA was extracted from cells using Trizol reagent (Takara, Beijing, China) and reverse transcribed using the reverse transcription kit (Takara, Beijing, China). RT‐qPCR was carried out with the SYBR Green Master Mix (Vazyme, Nanjing, China) on a CFX96 Touch System (Bio‐Rad, CA, USA). GAPDH was used as an internal control. Details of the primers used are provided in Table S6.

### 4.8 CCK‐8 Assay

1000 cells/well were seeded into 96‐well plates. Cell viability was continuously monitored by CCK‐8 assay (Beyotime, Shanghai, China) within 5 days. At the indicated time points, cells were cultured with a medium containing 10 µL CCK‐8 reagent at 37°C for 2 h. The absorbance was measured at 450 nm using a microplate reader.

### 4.9 Colony Formation Assay

Cells (500/well for KYSE30 and TE1, and 300/well for KYSE150) were seeded into 6‐well plates and cultured with a complete medium for 2 weeks. Following a rinse with PBS (Thermo Fisher Scientific, Waltham, USA), the cells were fixed with methanol for 30 min and then subjected to staining with crystal violet (Beyotime, Shanghai, China) for 20 min.

### 4.10 Co‐IP Assay

Cells were lysed in IP lysis buffer. The equal protein lysates were incubated with 30 µL Protein A/G magnetic beads (MCE, New Jersey, USA) and 3 µg antibodies or IgG at 4°C overnight. The beads‐bound proteins were washed with IP lysis buffer three times and eluted in 1 × SDS loading buffer (Leagene, Beijing, China) for Western blot, silver staining, or LC‐MS/MS analysis. Details of the antibodies used are provided in Table S4.

### 4.11 Silver Staining and LC‐MS/MS Analysis

Silver staining was conducted using the Fast Silver Stain Kit (Beyotime, Shanghai, China) according to the manufacturer's protocols. While protein identification was done by BGI Genomics (Shenzhen, China) based on LC‐MS/MS. Details of proteins with coverage > 0.02 are provided in Table S3.

### 4.12 IF Assay

Cells were rinsed with PBS, fixed with 4% paraformaldehyde, permeabilized with 0.1% Triton X‐100, and then blocked with 5% BSA. Primary antibodies were applied and incubated overnight at 4°C. On the second day, the cells were incubated with secondary fluorescent antibody for 1 h at room temperature in the dark, followed by nuclear staining with DAPI for 10 min. Images were acquired using the Olympus FluoView FV1000 confocal microscope (Olympus, Tokyo, Japan). Details of the antibodies used are provided in Table S4.

### 4.13 PLA

PLA was conducted using the Duolink in situ PLA kit (Sigma Aldrich, New Mexico, USA). Briefly, cells were fixed and incubated with primary antibodies (1:200 dilution), secondary PLA probes, and DAPI according to the manufacturer's protocols. Positive reactions were visualized using a 594 nm fluorescence label detection kit. Fluorescent signals arise from the hybridization between the two PLA plus and minus probes when the distance between the two antigens is less than 40 nm. Images were acquired by LSM880 with fast airyscan (ZEISS). Details of the antibodies used are provided in Table S4.

### 4.14 Glycolysis Analysis

For the lactate production analysis, the lactate concentration in the supernatants of cells cultured for 24 h was quantified enzymatically with a Lactic Acid Assay kit (Jiancheng Bio, Nanjing, China).

For the glucose uptake analysis, cells were harvested after 24 h of culture in a fresh medium. Intracellular glucose was quantified using the Glucose Uptake‐Glo Assay Kit (Promega, Wisconsin, USA).

For the Seahorse glycolysis analysis, 1 × 10^5^ cells/well were planted in a Seahorse XF96 cell culture plate. After adhering overnight, cells were incubated at 37°C in a CO_2_‐free incubator for 30 min before testing using a Glycolytic Rate Assay Kit of Agilent Seahorse XF (Agilent Technologies, California, USA). 1 h before measurement, the culture medium was replaced with Seahorse XF DMEM with pH 7.4 supplemented with 1 mM pyruvate, 2 mM glutamine, and 10 mM glucose. The drugs measuring the extracellular acidification rate (ECAR) were 50 µM 2‐DG, Rotenone, and 0.5 µM Antimycin A. All reagents were sourced from Sigma Aldrich (New Mexico, USA).

### 4.15 CHIP Assay

ChIP analysis was performed with the ChIP Assay Kit (Cell Signal Technology, Boston, USA). Briefly, cell lysates were prepared, and chromatin was fragmented into 200–1000 bp segments via sonication. Immunoprecipitation was conducted using anti‐H3K18la or anti‐IgG antibodies in conjunction with Protein A/G magnetic beads. Sequential washes were performed using low‐salt, high‐salt, lithium chloride (LiCl), and TE buffers to remove non‐specific interactions. Crosslinking was subsequently reversed to release the DNA fragments. Fold enrichment was determined by qPCR analysis and presented as a percentage of the input chromatin (Input%). Details of the primers used are provided in Table S6.

### 4.16 NRO Assay

NRO assay was performed as previously described [42]. Briefly, cell nuclei were isolated via cold trypsinization on ice and incubated with 60 µL transcription buffer mix containing 1 × transcription buffer (10 mmol/L Tris‐HCl, pH 8.3, 2.5 mmol/L MgCl2, 150 mmol/L KCl, and 2 mmol/L DTT), 100 U RNase inhibitor, 0.5 mmol/L BrUTP, 1 mmol/L ATP, 1 mmol/L GTP, 1 mmol/L CTP, and 0.5 mmol/L UTP. Transcription was carried out at 30°C for 30 mins. Nuclear RNA was then purified and quantified. BrUTP‐labeled nascent transcripts were immunoprecipitated, extracted, and subjected to reverse transcription followed by RT‐qPCR.

### 4.17 Flow Cytometric Analysis

Tumor tissues were excised, cut into small pieces, and re‐suspended in digestion buffer (RPMI‐1640 medium with 50U/mL type IV collagenase (Invitrogen, California, USA) and 20 U/mL DNase (Roche, Basel, Switzerland)). Tumors were digested at 37°C for 90 min and then passed through a 70 µm cell strainer. Flow samples were stained using 1 × 10^6^ cells. Gates were determined with fluorescence minus‐one (FMO) controls. Compensations and voltages were adjusted using single‐color controls. For intracellular staining of cytokines, T cells were stimulated with anti‐CD3/anti‐CD28 antibodies for 12 h. 10 µg/mL Brefeldin A (MCE, New Jersey, USA) was added into medium 4 h before harvesting. The harvested cells were subsequently fixed, permeabilized, and stained with the indicated antibodies. Samples were analyzed on a Fortessa flow cytometer (BD, New York, USA), and data were analyzed by FlowJo software. Details of the antibodies used are provided in Table S4.

### 4.18 T Cell Co‐Culture Experiments

Peripheral blood mononuclear cells (PBMCs) were obtained from healthy donors with approval from the Ethics Committee of GDPH. T cells were activated with plate‐bound anti‐CD3/anti‐CD28 antibodies in the presence of recombinant IL‐2 (100 U/mL) for 48 h. For the human T cell co‐culture assay, activated T cells were co‐incubated with tumor cells or culture supernatant derived from tumor cells. After 72 h of co‐culture, T cells were harvested and stained with the indicated antibodies.

### 4.19 Statistical Analysis

All experiments were conducted three times, and the data are presented as mean ± standard deviation (S.D.). Statistical analysis was performed using SPSS (version 25.0) or GraphPad Prism (version 8.0). The significance of differences was determined by a two‐tailed Student's *t*‐test or two‐way ANOVA. For survival analysis, the median was employed as the optimal cut‐point for NIPAL1 expression. The survival curve was analyzed by the Kaplan‐Meier method. A *p*‐value of less than 0.05 was defined as significant.

## Author Contributions

D.X., G.‐B.Q., and R.‐X.C. designed the study. R.‐X.C., X.‐D.M., S.‐D.X., A.‐T.M., and M.‐H.D. performed experiments and analyzed the data with help from J.‐H.W., W.‐T. Z., Z.H., S.‐H.X., J.‐L.D., J.‐M.T., and H.‐Y.Z. R.‐X.C., X.‐D.M., S.‐D.X., and A.‐T.M. draw the figures. R.‐X.C. and X.‐D.M. wrote the manuscript. X.‐S.B., G.‐B.Q., and D.X. conducted manuscript revision. All authors have read and approved the article.

## Conflicts of Interest

The authors declare no conflicts of interest.

## Supporting information




**Supporting File**: advs74775‐sup‐0001‐SuppMat.docx.

## Data Availability

The data that support the findings of this study are available from the corresponding author upon reasonable request.
