# Supplementary material for: NIPAL1 Drives a Metabolic‐Epigenetic Feedback Loop to Promote Lactate‐Mediated Immune Evasion in Esophageal Cancer
Source: Adv Sci (Weinh). 2026 Mar 12;13(29):e20055. doi: 10.1002/advs.202520055 (PMC13205849; doi:10.1002/advs.202520055)
Supplement: Supplementary file 1 — Supporting File: advs74775‐sup‐0001‐SuppMat.docx. [file ADVS-13-e20055-s001.docx]

**NIPAL1 Drives a Metabolic-Epigenetic Feedback Loop to Promote Lactate-Mediated Immune Evasion in Esophageal Cancer**

*Ri-Xin Chen^1,2,3#^, Xiao-Dan Ma^2#^, Shui-Dan Xu^2,4#^, Ao-Tian Mo^2#^, Min-Hua Deng^5#^, Jun-Han Wu^6^, Wei-Tao Zhuang^2,7^, Jin-Ling Duan^2,8^, Shu-Huan Xie^6^, Jin-Long Lin^2,9^, Peng Lin^9^, Ji-Ming Tang^1^, Hai-Yu Zhou^1^, Xiao-Song Ben^1^*, Gui-Bin Qiao^6^*, Dan Xie^2,8^**

^1^Department of Thoracic Surgery, Guangdong Provincial People’s Hospital (Guangdong Academy of Medical Sciences), Southern Medical University, Guangzhou 510080, China.

^2^State Key Laboratory of Oncology in South China, Guangdong Provincial Clinical Research Center for Cancer, Sun Yat-sen University Cancer Center, Guangzhou 510060, China.

^3^Medical Research Institute, Guangdong Provincial People’s Hospital (Guangdong Academy of Medical Sciences), Southern Medical University, Guangzhou 510080, China.

^4^Department of Radiology, Sun Yat-sen University Cancer Center, 510060, Guangzhou, Guangdong, China.

^5^Department of Urology, The First Affiliated Hospital of Guangzhou Medical University, Guangzhou, 510120, China.

^6^Department of Thoracic Surgery, Zhujiang Hospital, Southern Medical University, Guangzhou 510280, China.

^7^State Key Laboratory of Respiratory Diseases, National Clinical Research Center for Respiratory Disease, National Center for Respiratory Medicine, Department of Pulmonary and Critical Care Medicine, Guangzhou Institute of Respiratory Health, The First Affiliated Hospital of Guangzhou Medical University, Guangzhou 510120, China.

^8^ Department of Pathology, Sun Yat-sen University Cancer Center, Guangzhou 510060, China.

^9^Department of Thoracic Surgery, Sun Yat-sen University Cancer Center, Guangzhou 510060, China.

#These authors contributed equally: Ri-Xin Chen, Xiao-Dan Ma, Shui-Dan Xu, Ao-Tian Mo, and Min-Hua Deng.

*Correspondence: Dan Xie (e-mail: xiedan@sysucc.org.cn), Gui-Bin Qiao (e-mail: qiaoguibin@smu.edu.cn), or Xiao-Song Ben (e-mail: benxiaosong@gdph.org.cn).

**Supplemental Figures**

**Figure S1. NIPAL1 is upregulated in ESCC and promotes tumor cell proliferation.** (**A**) Western blot analysis of NIPAL1 protein expression in 12 ESCC tissues (T) and matched adjacent non-tumorous tissues (N). (**B**) Left, representative IHC staining for NIPAL1 in non-tumorous tissue (left) and ESCCs (right). Scale bar, 100 μm. Right, quantified H score of NIPAL1 in non-tumorous tissues (N) and ESCCs (T). (**C**) Kaplan-Meier survival analysis of overall survival (OS) in ESCC patients with low vs. high NIPAL1 expression (*n* = 52). (**D**) Western blot analysis of NIPAL1 protein expression in the indicated ESCC cell lines. (**E**) CCK-8 assay showing the viability of NIPAL1-knockdown ESCC cells. (**F**) Colony formation assay showing the proliferative capacity of NIPAL1-knockdown ESCC cells. Left, representative images. Right, quantification of colony numbers. (**G**) CCK-8 assay showing the viability of NIPAL1-overexpressing ESCC cells. (**H**) Colony formation assay showing the proliferative capacity of NIPAL1-overexpressing ESCC cells. Left, representative images. Right, quantification of colony numbers. (**I and J**) Subcutaneous xenograft model showing tumor formation of NIPAL1-knockdown (**I**) or NIPAL1-overexpressing (**J**) ESCC cells (*n* = 6). Left, subcutaneous tumor images. Right, quantification of subcutaneous tumor weights. Data represent mean ± S.D. from three independent experiments (**E**, **F**, **G**, **H**). The *P* value was determined by a two-tailed paired Student’s *t* test (**B**) or Log-rank test (**C**) or two-tailed unpaired Student’s t test (**F**, **H**, **I**, **J**), or two-way ANOVA (**E**, **G**).

**Figure S2. NIPAL1 interacts with LDHA and modulates its activity.** (**A**) Silver staining of proteins enriched by Flag-NIPAL1 in ESCC cells. (**B**) Peptide mapping analysis identifying LDHA in the Flag-NIPAL1 immunoprecipitates. (**C**) Western blot analysis of the indicated proteins in KYSE30 cells overexpressing LDHA WT or LDHA Y10F upon NIPAL1 knockdown.

**Figure S3. NIPAL1 modulates LDHA by facilitating HCK-LDHA interaction.** (**A**) Peptide mapping analysis identifying HCK in the Flag-NIPAL1 immunoprecipitates. (**B**) Protein levels quantified by Image J in sucrose gradient fractions from control and NIPAL1-knockdown cells. (**C**) Top, molecular docking of HCK with LDHA. Bottom, molecular docking of NIPAL1 with HCK and LDHA. (**D**) The schematic diagram representing the NIPAL1 WT and truncated mutant plasmids. (**E**) Western blot analysis of the indicated proteins in NIPAL1-overexpressing KYSE150 cells incubated with the indicated concentrations of A419259. (**F**) CCK-8 assay showing the viability of NIPAL1-overexpressing KYSE150 cells treated with A419259. (**G**) Colony formation assay showing the proliferative capacity of NIPAL1-overexpressing KYSE150 cells treated with A419259. Left, representative images. Right, quantification of colony numbers. Data represent mean ± S.D. from three independent experiments (**F**, **G**). The *P* value was determined by a two-tailed unpaired Student’s *t* test (**F**, **G**).

**Figure S4. NIPAL1 modulates lactate production independent of magnesium‐transport function.** (**A**) Western blot analysis of Flag-NIPAL1 immunoprecipitates showing the interaction between NIPAL1 and LDHA in control or NIPAL1-overexpressing KYSE150 cells treated with MgCl_2_. (**B**) Western blot analysis of HCK immunoprecipitates showing the binding of both NIPAL1 and LDHA to HCK in control or NIPAL1-knockdown KYSE30 cells treated with MgCl_2_. (**C and D**) Lactate levels in NIPAL1-overexpressing KYSE150 cells (**C**) or NIPAL1-knockdown KYSE30 cells (**D**) treated with MgCl_2_. Data represent mean ± S.D. from three independent experiments (**C**, **D**). The *P* value was determined by a two-tailed unpaired Student’s *t* test (**C**, **D**).

**Figure S5. *NIPAL1* was upregulated by tumor-derived lactate through histone lactylation.** (**A**) RT-qPCR analysis of *NIPAL1* mRNA levels in KYSE30 cells treated with 25 mM LA. (**B**) Western blot analysis of Flag-NIPAL1 immunoprecipitates showing lactylation levels of NIPAL1 in control or NIPAL1-overexpressing KYSE150 cells. Data represent mean ± S.D. from three independent experiments (**A**). The *P* value was determined by a two-tailed unpaired Student’s *t* test (**A**).

**Figure S6. NIPAL1 primarily resides on intracellular organelle membranes.** (**A**) Western blot analysis of the indicated proteins in different membrane fractions of ESCC cells. WC, whole cell. WM, whole membrane. PM, plasma membrane. OM, organelle membrane. (**B**) IF assays showing the colocalization of Flag-NIPAL1 with different membrane markers in KYSE150 cells. Nuclei were stained with DAPI (blue). Scale bar, 5 μm.

**Figure S7. Tumor-derived soluble lactate mediates CD8^+^ T cell suppression in a contact-independent manner.** T cell proliferation measured by CellTrace™ Violet (CTV) dilution via flow cytometry. T cells were labeled with CTV and cultured for 72 h with conditioned medium (CM) from control or NIPAL1-overexpressing ESCC cells. NIPAL1-overexpressing cells were pretreated for 24 h with GSK2837808A (20 µM), A419259 (1 µM), or A485 (1 µM) before CM collection. All CM were supplemented with 25 mM HEPES (pH 7.4) to minimize acidification effects. Left, representative histograms. Right, quantification of the proliferation index. Data are represented as mean ± S.D. from three independent experiments, and *P* values were determined by a two-tailed unpaired Student’s *t* test.

**Table S1.** **Relationship between NIPAL1 level and clinicopathological parameters of ESCCs**

| Variable | Number of cases | NIPAL1 expression | | *P* |
| --- | --- | --- | --- | --- |
|  |  | Low | High |  |
| Age (Year) |  |  |  | 0.139 |
| < 60 | 17 | 11 | 6 |  |
| ≥ 60 | 35 | 15 | 20 |  |
| Gender |  |  |  | ＞0.999 |
| Male | 42 | 21 | 21 |  |
| Female | 10 | 5 | 5 |  |
| Tumor location |  |  |  | 0.163 |
| Upper | 23 | 9 | 14 |  |
| Middle-lower | 29 | 17 | 12 |  |
| Histological grade (WHO) | |  |  | 0.092 |
| G1-2 | 30 | 18 | 12 |  |
| G3 | 22 | 8 | 14 |  |
| pT status |  |  |  | 0.026 |
| T1-T2 | 24 | 16 | 8 |  |
| T3 | 28 | 10 | 18 |  |
| Lymph node status | |  |  | 0.011 |
| No metastasis | 31 | 20 | 11 |  |
| Metastasis | 21 | 6 | 15 |  |
| ESCC, esophageal squamous cell carcinoma. | | | | |

**Table S2. Univariate and multivariate Cox regression analysis of different prognostic variables in patients with ESCC**

| Variable | Subset | Hazard ratio (95% CI) | *P* |
| --- | --- | --- | --- |
| Univariate analysis (*n* = 52) | | | |
| Age (Year) | < 60 *vs*. ≥ 60 | - | 0.870 |
| Gender | Male *vs*. Female | - | 0.301 |
| Tumor location | Upper *vs*. Middle-lower | 2.608 (1.127-6.036) | 0.015 |
| Histological grade (WHO) | G1-2 *vs*. G3 | 2.319 (1.015-5.296) | 0.036 |
| pT status | T1-2 *vs*. T3 | 2.508 (1.121-5.611) | 0.026 |
| pN status | N0 *vs*. N1 | 2.338 (1.002-5.454) | 0.031 |
| NIPAL1 expression level | Low *vs*. High | 3.114 (1.375-7.209) | 0.004 |
| Multivariate analysis (*n* = 52) | | | |
| Histological grade (WHO) | G1-2 *vs*. G3 | - | 0.276 |
| pT status | T1-2 *vs*. T3 | - | 0.136 |
| pN status | N0 *vs*. N1 | - | 0.508 |
| Tumor location | Upper vs. Middle-lower | 3.093 (1.032-5.431) | 0.042 |
| NIPAL1 expression level | Low *vs*. High | 3.093 (1.292-7.402) | 0.011 |
| ESCC, esophageal squamous cell carcinoma. | | | |

| **Table S3. List of candidates NIPAL1-interacting proteins identified by LC-MS/MS** | | | | |
| --- | --- | --- | --- | --- |
| Gene Name | Protein Q score | Molecular Weight | Unique Peptide | Coverage |
| DSP | 32.23782606 | 331568.7 | 9 | 0.046 |
| CANX | 23.14720309 | 67525.84889 | 6 | 0.1453 |
| RPS25 | 15.43146873 | 13733.69831 | 4 | 0.24 |
| DSG1 | 14.39007604 | 113675.8588 | 4 | 0.0677 |
| SLC25A3 | 11.57360154 | 40068.7367 | 3 | 0.1077 |
| HACD3 | 10.13201565 | 43131.55544 | 3 | 0.1188 |
| ENO1 | 10.13201565 | 47139.3184 | 3 | 0.0806 |
| **NIPAL1** | **8.752966445** | **44609.44704** | **3** | **0.122** |
| ESYT2 | 8.690429764 | 102293.9315 | 3 | 0.0369 |
| CSTA | 7.715734363 | 10999.66222 | 2 | 0.3061 |
| RPS13 | 7.715734363 | 17211.66624 | 2 | 0.1788 |
| VDAC2 | 7.715734363 | 31546.54592 | 2 | 0.102 |
| CASP14 | 7.715734363 | 27661.95119 | 2 | 0.095 |
| HSPA1B | 7.715734363 | 70009.03579 | 2 | 0.0499 |
| SEL1L | 7.715734363 | 88698.49548 | 2 | 0.0491 |
| DDX3Y | 7.715734363 | 73108.12007 | 2 | 0.0409 |
| H4C1 | 6.274148473 | 11360.3815 | 2 | 0.233 |
| SUCLG1 | 6.274148473 | 36226.84437 | 2 | 0.078 |
| HSPA9 | 6.274148473 | 73634.77131 | 2 | 0.0309 |
| TMSB4X | 6.154945928 | 5049.516165 | 2 | 0.4318 |
| H2AC4 | 6.071658398 | 14126.94964 | 2 | 0.2 |
| SERPINB12 | 6.071658398 | 46247.08621 | 2 | 0.0617 |
| UBC | 6.071658398 | 76991.53297 | 2 | 0.0219 |
| TUBA4A | 3.857867181 | 49892.36855 | 1 | 0.2679 |
| RPL39 | 3.857867181 | 6402.650395 | 1 | 0.1961 |
| IGKC | 3.857867181 | 11757.76816 | 1 | 0.1682 |
| APOC3 | 3.857867181 | 10845.4988 | 1 | 0.1616 |
| CALML5 | 3.857867181 | 15882.77126 | 1 | 0.1575 |
| RPS26 | 3.857867181 | 13007.08407 | 1 | 0.1304 |
| RPS24 | 3.857867181 | 15413.43159 | 1 | 0.1128 |
| IGKV2D-28 | 3.857867181 | 12948.45758 | 1 | 0.1083 |
| ONECUT2 | 3.857867181 | 54268.84044 | 1 | 0.1071 |
| S100A16 | 3.857867181 | 11793.9687 | 1 | 0.1068 |
| RPS20 | 3.857867181 | 13364.30823 | 1 | 0.0924 |
| MRPL14 | 3.857867181 | 15937.40754 | 1 | 0.069 |
| VKORC1L1 | 3.857867181 | 19822.67595 | 1 | 0.0625 |
| RPS19 | 3.857867181 | 16050.53475 | 1 | 0.0621 |
| RPL24 | 3.857867181 | 17767.89649 | 1 | 0.0573 |
| HSPA2 | 3.857867181 | 69977.86457 | 1 | 0.0563 |
| DIRAS2 | 3.857867181 | 22470.59761 | 1 | 0.0553 |
| NOL7 | 3.857867181 | 29408.52623 | 1 | 0.0545 |
| IGHA1 | 3.857867181 | 37630.63784 | 1 | 0.0482 |
| GAPDHS | 3.857867181 | 44472.78918 | 1 | 0.0441 |
| **HCK** | **3.857867181** | **59561.67358** | **1** | **0.0437** |
| PRSS1 | 3.857867181 | 26541.08856 | 1 | 0.0405 |
| ARG1 | 3.857867181 | 34713.29643 | 1 | 0.0373 |
| HNRNPA1L2 | 3.857867181 | 34204.26505 | 1 | 0.0312 |
| NAP1L1 | 3.857867181 | 45345.93287 | 1 | 0.0281 |
| PIGU | 3.857867181 | 50018.69085 | 1 | 0.0253 |
| ERLEC1 | 3.857867181 | 54823.28273 | 1 | 0.0248 |
| KATNAL1 | 3.857867181 | 55357.22599 | 1 | 0.0245 |
| BLMH | 3.857867181 | 52528.01898 | 1 | 0.0242 |
| DHCR7 | 3.857867181 | 54453.76212 | 1 | 0.0232 |
| SLC3A2 | 3.857867181 | 67951.72611 | 1 | 0.0222 |
| PKP1 | 3.857867181 | 82807.82883 | 1 | 0.0201 |
| S100A14 | 2.816474496 | 11654.76049 | 1 | 0.1058 |
| RPL10L | 2.816474496 | 24502.71265 | 1 | 0.0514 |
| ZNF706 | 2.416281291 | 8492.447015 | 1 | 0.1579 |
| SCGB1D2 | 2.416281291 | 9918.341425 | 1 | 0.1 |
| RBIS | 2.416281291 | 11449.34407 | 1 | 0.09 |
| RPL36AL | 2.416281291 | 12460.81785 | 1 | 0.066 |
| NME5 | 2.416281291 | 24220.61126 | 1 | 0.0425 |
| **LDHA** | **2.416281291** | **36665.36034** | **1** | **0.0271** |
| RPS23 | 2.213791217 | 15797.71686 | 1 | 0.0769 |
| MBOAT7 | 2.213791217 | 52730.26299 | 1 | 0.0254 |
| TAS2R3 | 2.078624768 | 35891.14498 | 1 | 0.0411 |
| SURF4 | 2.078624768 | 30373.78284 | 1 | 0.0372 |

| **Table S4. List of antibodies** | | | | | |
| --- | --- | --- | --- | --- | --- |
| Name | Manufacturer | Lot. No. | Source | Application | RRID |
| Anti-NIPAL1 | LifeSpan | LS‑C164878 | Rabbit | WB | AB_3714828 |
| Anti-NIPAL1 | Sigma-Aldrich | HPA036765 | Rabbit | IHC | AB_10672366 |
| Anti-LDHA | Proteintech | 19987-1-AP | Rabbit | WB/IP/  IF/PLA | AB_10646429 |
| Anti-p-LDHA (Y10) | Affinity | AF7172 | Rabbit | WB/IHC | AB_2843612 |
| Anti-HCK | Santa Cruz | sc-101428 | Mouse | WB/IP/  PLA | AB_2115144 |
| Anti-Flag | Cell Signal Technology | 8146 | Mouse | WB/IP/IF | AB_3714831 |
| Anti-Myc | Cell Signal Technology | 2276 | Mouse | WB | AB_331783 |
| Anti-His | Cell Signal Technology | 2365 | Rabbit | WB | AB_2115720 |
| Anti-L-Lactyllysine | PTM Bio | PTM-1401RM | Rabbit | WB | AB_2942013 |
| Anti-H3K9la | PTM Bio | PTM-1419RM | Rabbit | WB | AB_3076695 |
| Anti-H3K18la | PTM Bio | PTM-1406RM | Rabbit | WB/  CHIP/  IHC | AB_2909438 |
| Anti-H4K12la | PTM Bio | PTM-1411RM | Rabbit | WB | AB_2941896 |
| Anti-H3 | Proteintech | 17168-1-AP | Rabbit | WB | AB_2716755 |
| Anti-GAPDH | Proteintech | 60004-1-Ig | Mouse | WB | AB_2107436 |
| Anti-Na^+^/K^+^ ATPase | Abcam | ab76020 | Rabbit | WB/IF | AB_1310695 |
| Anti-TOMM20 | Proteintech | 11802-1-AP | Rabbit | WB/IF | AB_2207530 |
| Anti-Calnexin | Proteintech | 10427-2-AP | Rabbit | WB/IF | AB_2069033 |
| Anti-Giantin | Abcam | ab317612 | Rabbit | WB/IF | AB_3714833 |
| Anti-CD25-FITC | BioLegend | 985812 | Mouse | FC | AB_2922662 |
| Anti-CD69-APC/cy7 | BioLegend | 985206 | Mouse | FC | AB_2922660 |
| Anti-CD8-PE | BioLegend | 980902 | Mouse | FC | AB_2616623 |
| Anti-IFNγ-PB | BioLegend | 502522 | Mouse | FC | AB_893525 |
| Anti-GZMB-FITC | BioLegend | 515403 | Mouse | FC | AB_2114575 |
| Anti-CD274-PE | BioLegend | 329706 | Mouse | FC | AB_940368 |
| Anti-CD45-APC | BioLegend | 103111 | Rat | FC | AB_312976 |
| Anti-CD3-Bv421 | BioLegend | 100228 | Rat | FC | AB_2562553 |
| Anti-CD8-Bv605 | BioLegend | 100744 | Rat | FC | AB_2562609 |
| Anti-PD-1-FITC | BioLegend | 135213 | Rat | FC | AB_10689633 |
| Anti-TIM-3-APC/Fire 750 | BioLegend | 119737 | Rat | FC | AB_2810367 |
| Anti-TIGIT-PE | BioLegend | 142103 | Rat | FC | AB_10933258 |
| Anti-CD3-FITC | BioLegend | 100203 | Rat | FC | AB_312660 |
| Anti-GZMB-Bv421 | BioLegend | 396414 | Rat | FC | AB_2810603 |
| Anti-IFN-γ-PE | BioLegend | 505807 | Rat | FC | AB_315401 |
| Anti-TNF-α-PE/cy7 | BioLegend | 506323 | Rat | FC | AB_2204356 |

| **Table S5. List of shRNA sequences** | | |
| --- | --- | --- |
| Name | Sequence (5'-3') |  |
| shCTRL | ACAGAAGCGATTGTTGATC | |
| NIPAL1-shRNA-1 | GCCAGTTTACAAACATCCGCT | |
| NIPAL1-shRNA-2 | GCTAAGAAAGAAGCCGTCTCT | |

| **Table S6. List of primers** | |  |
| --- | --- | --- |
| Primer name | qPCR primer sequence (5’→3’) | |
| *NIPAL1*-Forward | GCTGGCTGTACTTGTGCTTT | |
| *NIPAL1*-Reverse | AGATGGCAGAGCAAGTCACT | |
| *NIPAL1* promoter-Forward | GCTGGCTGTACTTGTGCTTT | |
| *NIPAL1* promoter-Reverse | AGATGGCAGAGCAAGTCACT | |
| *GAPDH*-Forward | TGCACCACCAACTGCTTAGC | |
| *GAPDH*-Reverse | GGCATGGACTGTGGTCATGAG | |
